# 3D Imaging in Unilateral Primary Pulmonary Hypoplasia in an Adult: A Case Report

**DOI:** 10.1155/2011/659586

**Published:** 2011-10-19

**Authors:** Aristida Georgescu, Crinu Nuta, Simona Bondari

**Affiliations:** ^1^Emergency Clinical County Hospital of Craiova, Prima Medical SRL Craiova, No. 1, Tabaci Street, 200642 Craiova, Romania; ^2^Clinical Infectious Diseases and Pneumo-ftysiology Hospital “V. Babes” Craiova, No. 126, Calea Bucuresti Street, 200515 Craiova, Romania

## Abstract

Unilateral primary pulmonary hypoplasia is rare in adulthood (UPHA); it is characterized by a decreased number of bronchial segmentation and decreased/absent alveolar air space. Classical chest X-ray may be confusing, and the biological tests are unspecific. We present a case of UPHA in a 60-year-old female, smoker, with 3 term normal deliveries, who presented with late recurrent pneumonias and bronchiectasis-type symptomathology, arterial hypertension, and obesity. Chest X-rays revealed opacity in the left lower pulmonary zone, an apparent hypoaerated upper left lobe and left deviation of the mediastinum. Preoperatory multidetector computer tomography (MDCT) presented a small retrocardiac left lung with 5-6 bronchial segmentation range and cystic appearance. After pneumonectomy the gross specimen showed a small lung with multiple bronchiectasis and small cysts, lined by hyperplasic epithelium, surrounded by stromal fibrosclerosis. We concluded that this UPHA occurred in the 4–7 embryonic weeks, and the 3D MDCT reconstructions offered the best noninvasive diagnosis.

## 1. Introduction

The development anomalies of the lung between the 4th and 24th gestational weeks may cause functional damage usually discovered in newborns and infants, and it can be rarely present in adulthood [1]. Practically, the earlier the anomaly is present, the branching of the tracheal-bronchial tree is reduced [2]. 

The degree of lung involvement was classified into three groups by Boyden [3].

- Pulmonary agenesis: carina, main bronchi, lung tissue, and vascular structures are absent;
- Pulmonary aplasia: a pouch-like, blind-ending main bronchus and carina are present;
- Pulmonary hypoplasia: the destroyed bronchial structures cause maldevelopment in alveolar tissue, lung tissue is seen as a mediastinal structure.

Pathologically, the hypoplastic lung has reduced lung weight, fewer generations of airways, and hypoplasia of the corresponding pulmonary arteries. Epithelial differentiation is delayed, and surfactant deficiency is associated. In pulmonary hypoplasia, a mediastinal shift to the side of a homogenous density may be depicted, with compensatory herniation of the uninvolved lung.

Etiologically, the pulmonary hypoplasia can be classified as primary, if there is not any obvious cause of hypoplasia; respiratory distress can be seen immediately after birth, and the higher mortality rate is generally due to severe pulmonary circulatory abnormalities. If there are other fetal and maternal anomalies, the pulmonary hypoplasia is classified as secondary; space-occupying lesions in the chest (including diaphragmatic hernia), developmental anomalies of the chest wall, urogenital and neuromuscular diseases can be associated, and they represent supplementary risk factors for early complications and death [4]. 

Clinical presentation in adult is highly variable, depending in large measure on a history of smoking and repeated respiratory infections. The almost asymptomatic cases with long survival in adulthood are explained by compensatory hypertrophy of the contra lateral lung filling of the ipsilateral hemithorax, as in pneumonectomy; the best survival is for the left lung hypoplasia, because of the good compensatory hypertrophy of the larger right lung.

## 2. Case Presentation

This paper presents a case of UPHA in a 60-year-old female, whose diagnosis was misinterpreted up to 58 years old. 

As history, the patient was asymptomatic at birth and in the childhood; she presented a pneumopathy at 19 years old, and the chest X-rays revealed a dense opacity in the left lower pulmonary zone extended to the pleural basal sinuses, and 3-month-tuberculostatic preventive therapy was applied. The patient had good condition during the most part of life, with 3 term pregnancies finished with normal deliveries. She supported also some surgical treatments without complications: ovarian cystectomy at 30 years old and laparoscopic cholecistectomy at 54 years old. The occasionally chest X-rays were interpreted as left basal pleural thickening and chronic pneumopathy, sequel of the previous infections. 

At presentation, the patient of 58 years old, smoker for 20 years, with obesity and arterial hypertension with the mean blood pressure 180/100 mm Hg, accused symptoms suggesting recurrent bacterial “broncho pneumonias” with high fever, chills, and cough with mucous-purulent expectoration, left chest pain, breathlessness on exertion and wheezing. 

The biological tests revealed the white cells blood count with infectious formula, and the erythrocyte sedimentation rate (ESR) raised up to 46 mm/hr and 68 mm/2 hr. The electrocardiogram presented a left cardiac axis deviation with QRS +50°, microvoltage of the QRS complexes in the standard deviations, and negative T waves in V3–V6. The abdominal and pelvic ultrasonography did not reveal any congenital abnormality and confirmed the status after cholecistectomy. 

**Figure 1:**
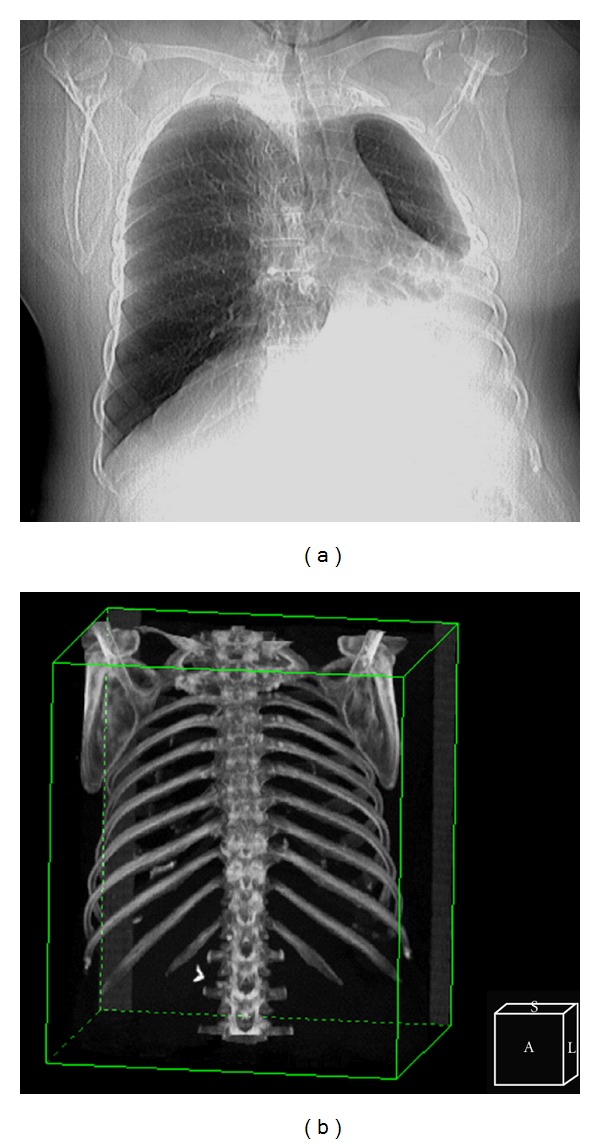
The radiological aspect could suggest a left basal pachypleuritis and a left lower lobe atelectasis, with ipsilateral mediastinal deviation and right lung herniation (a); 3D bone reconstruction revealed a symmetrical thoracic cage, discordant with an acquired retractile pleural-pulmonary pathology (b).

The posterior-anterior chest roentgenogram offered an erroneous diagnosis, presenting an apparently opacity in the left lower pulmonary zone, a hypoaerated upper left lobe and left deviation of the mediastinum, as we present in this CT scanogram (Figure 1(a)). We can mention that despite the apparent atelectasis of the left lung, there is not a significant retraction of the left hemithorax, such as in the postnatal acquired pathology (Figure 1(b)).

**Figure 2:**
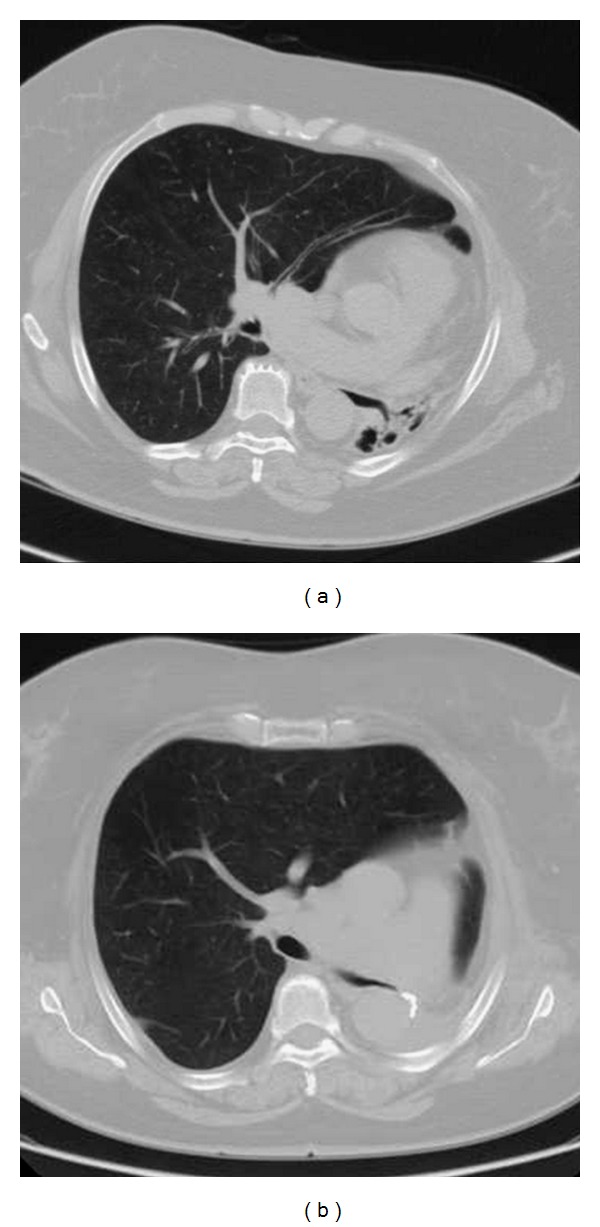
The axial scans in lung window imaging reconstruction before (a) and after (b) the left pneumonectomy.

The MDCT demonstrated a small left lung consisting of multiple cysts, a hyperinflation of the right lung with left side herniation mostly in the upper zone and left mediastinal deviation. The axial scans in lung and mediastinal windows images (Figures 2 and 5(a)) were considered essential for the counting of the bronchial segmentation range, but not enough for the understanding of the anatomical compensatory development of the right lung and of the mediastinal structures.

**Figure 3:**
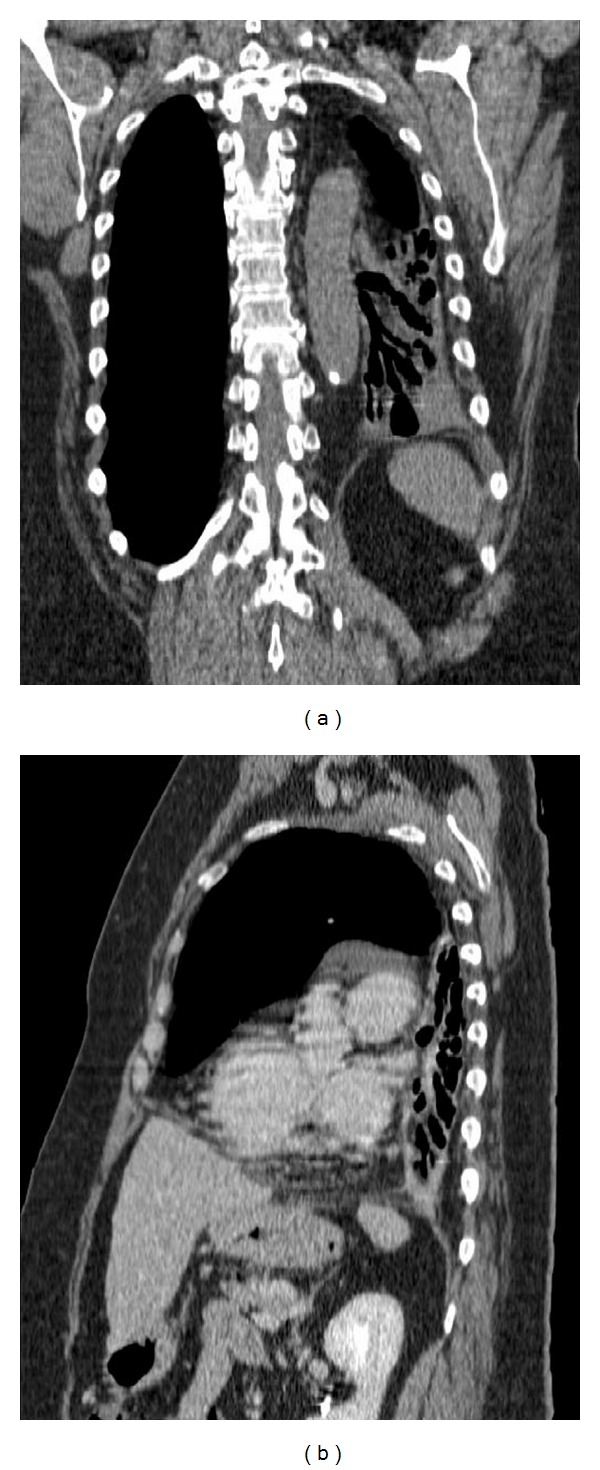
The coronal (a) and sagittal (b) reconstructions in mediastinal window imaging allow a better understanding of the anatomical position of the supra- and underdiaphragmatic organs.

Indeed, there was not only an ipsilateral mediastinal deviation, but also we admit a special adapted development of the functional thoracic organs and tissues; the multiplanar reconstructions demonstrated the depression of the right hemidiaphragm that vas balanced to allow the right lung hypertrophy (not only a hyperinflation) with a normal position of the left hemidiaphragm (Figures 3(a) and 3(b)). 

**Figure 4:**
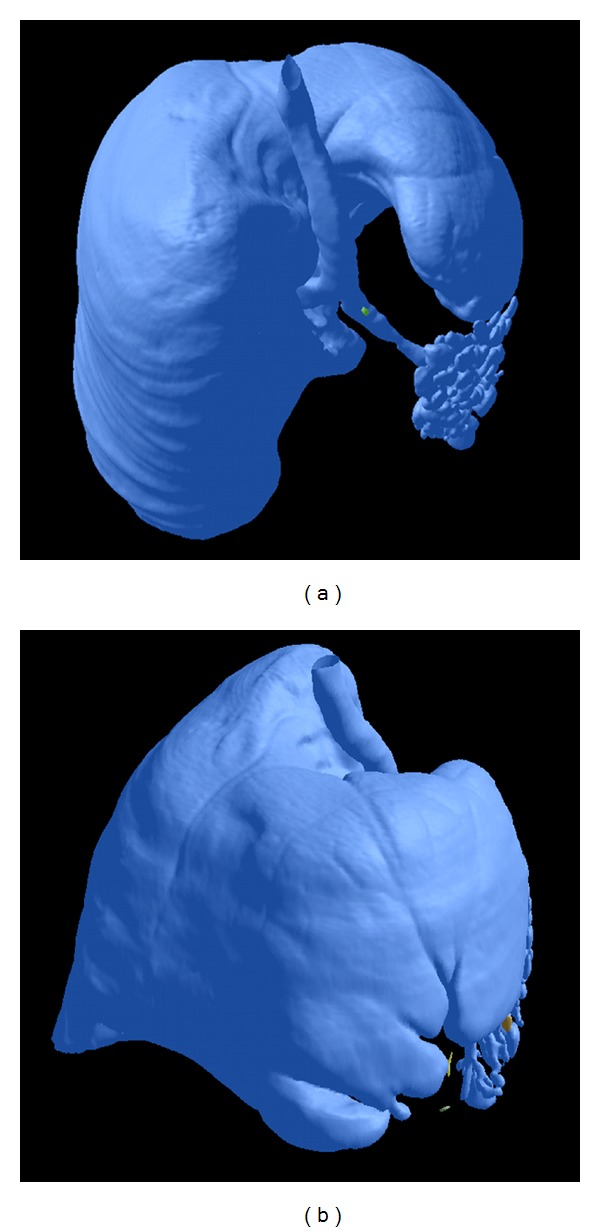
3D Reconstructions in the “surface” mode visualize both the hypoplastic dysplastic left lung, with multicystic architecture of small range segmentation (a), and the right hypertrophic lung with the pleural fissures and the abnormal lobar orientation (b). The upper lobe is in the left-posterior location, the middle lobe is in the left-anterior position, and the lower lobe is emptying the retrosternal space and the right hemithorax. The mediastinal space is reduced and posterior located, surrounded by the “horseshoe” right lung.

The best understanding of the anatomical relations was offered by the volumic reconstructions of the MDCT acquisitions, using dedicated medical imaging software that offered a superior computer-assisted diagnostics (CAD) platform [5]; indeed, 3D views can reveal abnormalities that may otherwise be overlooked by radiologists and other medical professionals. In this case, the right lung development was dimorphic, the lobes were oriented in different directions to empty the thoracic volume, the pleural fissures and the right bronchial tree are not proportional, while the left lung appears as a cluster of small cysts connected to the lobar bronchi, without any alveolar structure. For the reconstructions we maintained conventionally the left lobe on the left-side of the screen in the posterior-anterior view, for better recognition when comparing with the axial and the coronal scans (Figure 4). 

**Figure 5:**
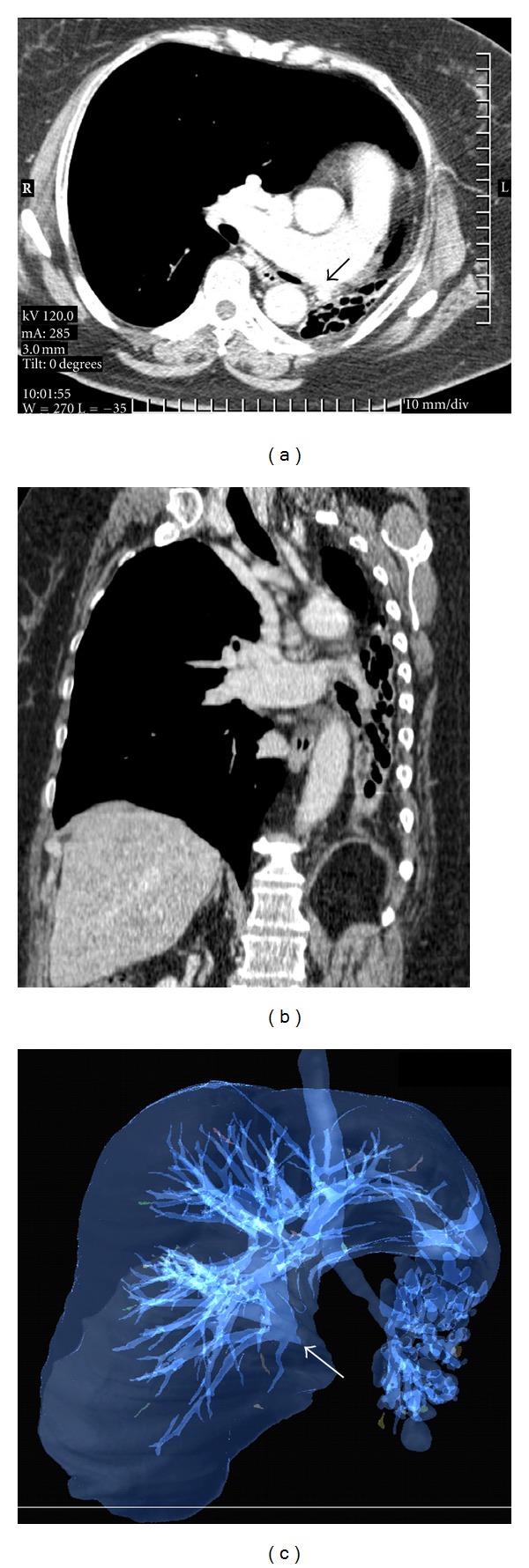
Contrast MDCT illustrates the apparent blind-ended hypoplastic left pulmonary artery (black arrow) and the dilated right pulmonary artery (a); coronal reconstruction visualizes the hypoplastic left pulmonary artery (b), and 3D reconstruction in the “transparency” mode (c) demonstrates the main bronchial orientation, the cystic structure of the left lung and the presence of the functional vascular architecture only in the developed lung (white arrow).

Moreover, in the “transparency” reconstruction mode, we can see the large development and the special orientation of the right pulmonary vessels, while the hypoplastic lung has no salient vasculature (Figure 5). 

**Figure 6:**
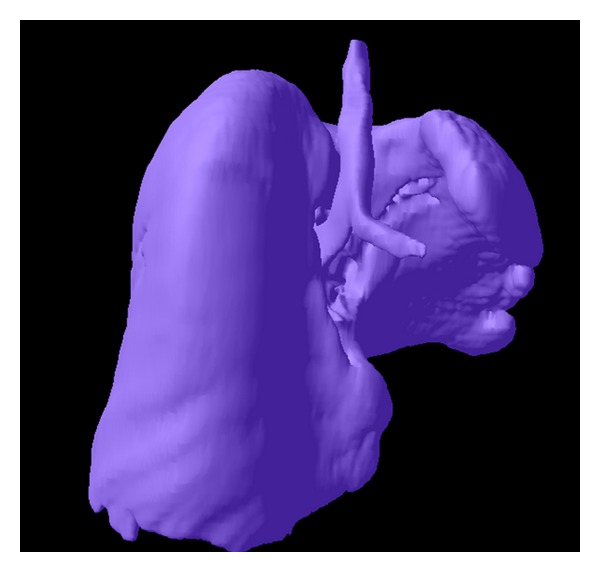
Postoperatory MDCT with 3D view reconstruction showing the blind-ended left main bronchia and the compensatory hyperplasic right lung.

The surgical treatment was decided, and a left pneumonectomy was performed without complication (Figure 6), and the gross specimen examination revealed a small, apparently retracted densified lung, with low elasticity and abolished crepitus; on section many bronchiectasis were mucus-filled, some with a cystic appearance. 

The microscopic examination of the surgical specimen showed a pulmonary structure characterized by reshuffle areas with dilated bronchi with thick walls lined by hyperplasic epithelium, surrounded by stromal fibrosclerosis and vascular stasis. There were not any alveoli or bronchioli.

The followup exams revealed after 2 years a pulmonary function test with severe restriction, but in this interval there were no more respiratory infectious signs. The white cells blood count presented normal value of 6.4 · 10^3^/*μ*L, while the red cells blood count sketched compensatory changes with an elevated VEM of 96.3 fL (normal 80.00–95.00 fL), hemoglobin of 15.5 g/dL (normal 12–15 g/dL); the erythrocyte sedimentation rate (ESR) decreased toward 24 mm/hr and 42 mm/2 hr.

## 3. Discussion

From the clinical point of view, most of the pulmonary hypoplasia cause severe respiratory failure; in adult individuals the diagnosis is difficult, since there are very few relevant symptoms and signs. Based on the anatomical aspects correlated with the embriological lung development (Table 1), we concluded that this UPHA occurred in the first 7–10 weeks of fetal age, allowed a contralateral pulmonary hypertrophy with good compensation, but the dysplastic lung determined repeated bronchitis followed by unuseful drugs treatments due to the false X-ray diagnostic. 

MDCT with the “classical” axial views and especially the multiplanar and the 3D reconstructions allowed the more accurate diagnostic, practically the angiography becoming unnecessary. Indeed, in the left hypoplastic lung, there was no more than 5-6 range bronchial segmentation. This early malformation was simultaneous with the left pulmonary artery extremely hypoplasia, concordant with the observation of Kurkcuoglu et al. [1] who presented a left pulmonary hypoplasia and showed by pulmonary arteriography a normal coursing of the right, dilated pulmonary artery and the absence of the ipsilateral (left) pulmonary artery. This early event explains the absence of the alveoli in the pathologic report and also the overextension and the reorientation in the whole thoracic remnant space of the right lung, so the symmetry of the thoracic cage was preserved. 

The absence of the associated embryological pathology argues the primary lung hypoplasia, with a better life prognostic than the secondary pulmonary hypoplasia; the left-sided involvement allowed a good survival despite of the risk factors (smoking, obesity, and arterial hypertension), due to the larger compensatory possibility of the right lung composed of 3 lobes [6]. 

**Figure 7:**
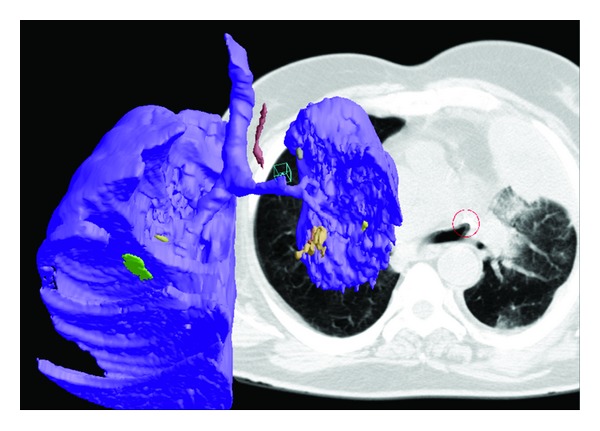
Small left lung after lobectomy and atelectasis of the remnant lobe due to alveolar collapse by pleural fluid collection and alveolar consolidation. There is no significant mediastinal shift toward the involved side, and the branches of the left pulmonary artery are present.

Some differential diagnosis must be discussed, because of the rare incidence of this pathology, and the first differential diagnosis is a *secondary (acquired) pneumopathy with noncongenital bronchiectasis*, as was considered the wrong initial diagnosis based on the late history, the clinical exam, and the chest roentgenograms; there is the small range of bronchial segmentation in a small, hypoplastic lung, while alveoli are always present with or without consolidation in the secondary small lung (pseudo-hypoplasia) (Figure 7). 

*Cystic adenomatoid malformation (CAM)* is a developmental hamartomatous abnormality of the lung, with adenomatoid proliferation of cysts resembling bronchioles. CAM accounts for 25% of all congenital lung malformations [6]. By contrarily to UPHA, the radiographic pattern appears as an expansile soft-tissue mass containing multiple air-filled cystic masses of varying size and shifting of the mediastinum. The involved lung may appear honeycombed or spongy, but occasionally, 1 large cyst may overshadow the others. The radiographic depiction of a solid or cystic mass on one side of the thorax was considered utile for the diagnosis, but we consider MDCT or chest Magnetic Resonance Imaging (MRI) with multiplanar and 3D reconstructions using the performing soft programs disposable nowadays are the methods of choice to characterize this abnormalities; lesions that may appear to have resolved on radiography can still be identified on the chest CT scan. 

CAM is rare in adulthood and could be confused with UPHA or secondary bronchial pathology; Vicidomini et al. [7] reported a case in a 62-year-old male, who presented with recurrent bacterial pneumonias and breathlessness on exertion. Other reports described the clinical and MDCT image characteristics of CAM of the lung in adults [8], with a multiloculated cystic mass in one lobe with normal vascular images.

*Pulmonary sequestration* accounts for 6% of all congenital lung malformations and mostly occurs in the lower lobes, characterized by a bronchopulmonary mass without a normal bronchial communication and with normal or anomalous vascular supply. The similarities with pulmonary hypoplasia are recurrent respiratory problems in the same anatomic location, associated anomalies including diaphragmatic hernia and eventration. The diagnostic is based on contrast MDCT, which illustrates the unusual solid attenuation without bronchial connection and the anomalous vascular supply. 

*Congenital lobar emphysema* (CLE) could mimic the compensatory overinflation of the contralateral lung in UPHA. Causes of the CLE include intrinsic absence or abnormality (bronchomalacia) of cartilaginous rings or external compression by a large pulmonary artery, which is equally present in UPHA. Hyperexpansion of a pulmonary lobe is present after birth when, with negative inspiratory pressure, air can enter the lung. However, the air cannot exit easily because positive pressure causes the softened airway to collapse. The remaining normal lung is then compressed, the pulmonary function resulting with severe obstruction illustrated on Spirometry. Moreover, the CLE primarily involves the upper lobes and most patients with congenital lobar emphysema present before 6 months of life. On MDCT the involved lobe and its vascularity can be easily outlined as compared to normal lung parenchyma, and the contralateral lobe presents a normal architecture of the bronchial and of the vascular tree.

## 4. Conclusion

Although congenital lung malformations are rare, they are important disorders because they may lead to considerable morbidity and mortality (infection, hemorrhage, and respiratory failure). Prognosis depends on the size of the lesion, the degree of functional impairment, and the associate congenital malformations. 

In UPHA, both the bronchial tree and the pulmonary vascularity are simultaneously affected and the range of the bronchial tree could determine the gestational age of the malformation occurrence. 

UPHA may remain asymptomatic or misinterpreted for many years; the unspecific clinical signs and the classical chest X-rays may lead to late diagnosis of the pulmonary hypoplasia, primary or secondary. Failure to recognize a malformation may lead to inappropriate treatment and delayed surgical intervention such as in this case. In some cases, the diagnosis even after computed tomography with the usual protocol was précised postoperatory: Ayadi-Kaddour et al. [9] reported 2 from 3 cases and Oh et al. mentioned 1 from 7 cases with late confirmation [8]. Some authors consider the late diagnosis of UPHA, possibly because the anomalies observed are attributed to old infections, and the clinical presentation is highly variable, depending in large measure on a history of smoking and repeated respiratory infections [3]. 

Chest MDCT is at present the diagnostic tool of choice, allowing the computed aided diagnosis techniques, with the best noninvasive characterization of the anatomy.

## Figures and Tables

**Table 1:** Development of the lung (based on Hislop [2]).

Embryonic stage: 4–7 weeks	Bronchial segmentation: (i) up to 10 generations	*Extrapulmonary artery: 34 days * *Lobular arteries: 44 days*

Pseudoglandular: 5–17 weeks	Bronchi, bronchioli, respiratory bronchioli: (i) up to 18 generations	*Preacinar arteries: 5–17 weeks*

Canalicular: 16–23 weeks	Respiratory bronchioli, alveolar ducts: (i) up to 22 generations	*Intra-acinar arteries: 18–25 weeks, alveolar duct arteries*

Maturation stage	Sacullus: (i) up to 23 generations; alveoli	*Alveolar duct arteries: 25 weeks–18 months postnatal * *Alveolar capillaries: 36 weeks–18 years*
